# Optimization of Postural Control, Balance, and Mobility in Children with Cerebral Palsy: A Randomized Comparative Analysis of Independent and Integrated Effects of Pilates and Plyometrics

**DOI:** 10.3390/children11020243

**Published:** 2024-02-15

**Authors:** Ragab K. Elnaggar, Rodrigo Ramirez-Campillo, Alshimaa R. Azab, Saud M. Alrawaili, Mshari Alghadier, Mazyad A. Alotaibi, Ahmed S. Alhowimel, Mohamed S. Abdrabo, Mohammed F. Elbanna, Ahmed M. Aboeleneen, Walaa E. Morsy

**Affiliations:** 1 Department of Health and Rehabilitation Sciences, College of Applied Medical Sciences, Prince Sattam Bin Abdulaziz University, Al-Kharj 11942, Saudi Arabia; 2 Department of Physical Therapy for Pediatrics, Faculty of Physical Therapy, Cairo University, Giza 12613, Egypt; 3 Exercise and Rehabilitation Sciences Institute, Faculty of Rehabilitation Sciences, Universidad Andres Bello, Santiago 7591538, Chile; 4 Department of Basic Sciences, Faculty of Physical Therapy, Cairo University, Giza 11432, Egypt; 5 Department of Medical Rehabilitation Sciences, College of Applied Medical Sciences, Najran University, Najran 61441, Saudi Arabia; 6 Department of Physical Therapy, Faculty of Medical Rehabilitation Sciences, King Abdulaziz University, Jeddah 21589, Saudi Arabia; 7 Department of Physical Therapy, College of Applied Medical Sciences, Jazan University, Jazan 45142, Saudi Arabia

**Keywords:** children, spastic cerebral palsy, rehabilitation, exercise therapy, strength training, physical conditioning, motor function

## Abstract

The paradigm of comprehensive treatment approaches for children with cerebral palsy has gained traction, prompting clinicians to deliberate between independent and integrated treatment delivery. However, this decision-making process is often hindered by the dearth of empirical evidence available to inform optimal therapeutic strategies. This study, therefore, sought to compare the effects of Pilates-based core strengthening (PsCS), plyometric-based muscle loading (PlyoML), and their combination on postural control, balance, and mobility in children with unilateral cerebral palsy (ULCP). Eighty-one children with ULCP (age: 12–18 years) were randomized to PsCS (*n* = 27), PlyoML (*n* = 27), or a combined intervention (*n* = 27; equated for total sets/repetitions) group. The three interventions were applied twice/week over 12 successive weeks. Postural control (directional and overall limits of stability—LoS), balance, and mobility (Community Balance and Mobility Scale—CB&M; Functional Walking Test—FWT; Timed Up and Down Stair test—TUDS) were assessed pre- and post-intervention. The combined group exhibited greater increases in directional LoS compared to PsCS and PlyoML including the backward (*p* = 0.006 and 0.033, respectively), forward (*p* = 0.015 and 0.036, respectively), paretic (*p* = 0.017 and 0.018, respectively), and non-paretic directions (*p* = 0.006 and 0.004, respectively)], and this was also the case for overall LoS (*p* < 0.001 versus PsCS and PlyoML). In addition, the combined group displayed greater improvements compared to the PsCS and PlyoML groups regarding CB&M (*p* = 0.037 and *p* = 0.002, respectively), FWT (*p* = 0.012 and *p* = 0.038, respectively), and TUDS (*p* = 0.046 and *p* = 0.021, respectively). In conclusion, the combined PsCS and PlyoML exercise program promotes considerably greater improvements in postural control, balance, and mobility compared to unimodal training in children with ULCP.

## 1. Introduction

Cerebral palsy (CP) is a non-progressive neurodevelopmental disorder characterized by impairments in movement and posture, stemming from injury or anomalous brain development. It stands as a prominent motor disability encountered during childhood, with a prevalence ranging from 1.5 to over 4 per 1000 live births on the global scale [1,2]. Unilateral CP (ULCP), a specific variant of CP, manifests as motor impairments predominantly affecting one side of the body. This impairment pattern is typically attributed to underlying damage or developmental abnormalities within the contralateral hemisphere of the brain. Epidemiological data corroborate the considerable prevalence of ULCP, accounting for approximately 33–39% of the overall CP population [3]. CP exhibits a wide spectrum of motor impairments, including spasticity, muscle weakness, postural control deficits, and disturbances in balance and gait [1,4]. These multifaceted motor limitations significantly impact the functional capacities of children with CP, compromising their independence, curtailing their participation in everyday activities, and significantly diminishing their overall quality of life [5].

One of the primary impairments in children with unilateral CP (ULCP) is postural control dysfunction [6]. Numerous factors may impact postural control, either individually or collectively, such as muscle spasticity/paresis, contractures, biomechanical alignment alterations, and sensory–perceptual deficits [7]. Children with ULCP have a limited ability to employ reactive and/or anticipatory postural control strategies [8,9]. The two basic mechanisms required for postural control have been shown to be affected in children with ULCP; the sensory organization mechanism (through which the central nervous system integrates the somatosensory, visual, and vestibular systems) and the motor adjustment mechanism (wherein coordinated musculoskeletal responses are used to maintain postural stability) [10,11]. Children with ULCP have trouble coordinating the optimum sequential activation of postural muscles, which is notably evident during functional activities [12]. As a result of this impairment, significant functional limitations are imposed. Enhancing postural control and optimizing functional performance in children with ULCP are the overriding goals of physical rehabilitation. In general, there are a variety of exercise-based interventions for children with ULCP to enhance their postural control and functionality; Pilates-based core strengthening (PsCS) and plyometric-based muscle loading (PlyoML) [13,14,15,16,17], among others, have been found to help with postural control and functional performance.

The PsCS is a training model that focuses on increasing muscle strength, endurance, and flexibility while maintaining spine stability. Exercises typically entail controlled, precise movements that target the muscles in the abdomen, back, hips, and thighs. These are frequently carried out on a mat, though they can also be done using specialized equipment [17]. The fundamental principles that underpin PsCS practice include concentration; focusing on the entire body to ensure smooth movements (centering); beginning movement from the core and flowing out to the limbs (control); performing slow, controlled movement with an emphasis on postural alignment (flow); a smooth transition between movements (precision); moving in a precise and exact way; and breathing (synchronizing breathing with movements) [18]. Prior studies have upheld the clinical implications of using PsCS either in a single mode or in combination with neurodevelopmental therapy (NDT) in selected samples of children with ULCP who can stand and walk independently. A recent randomized controlled study by Adıguzel and Elbasan [13] found greater gains in trunk, postural control, balance, and gait performance after an 8-week PsCS program compared to NDT. Abd-Elfattah et al. [14] noted that PsCS was an effective add-on therapy (to 10 weeks of NDT) for enhancing balance and motor function in children with ULPC. Furthermore, an earlier case report by Dos Santos et al. [17] observed that children with ULCP improved their muscle strength and postural control after eight weeks of PsCS.

The PlyoML, also referred to as the stretch–shortening cycle exercise, is a training method that involves a rapid eccentric (lengthening) contraction of a muscle immediately followed by a rapid concentric (shortening) contraction of the same muscle, resulting in a powerful explosive movement [19,20]. The PlyoML enhances concentric power output via stretch–shortening cycle mechanisms that involve neural factors (such as pre-contraction potentiation, stretch reflex activation, and motor unit recruitment), mechanical factors (i.e., storage and release of elastic energy), and contractile factors (like optimizing the length of sarcomeres and increasing the number of active cross-bridges) [20,21]. The PlyoML, although not a new concept, has recently grabbed the attention of pediatric rehabilitation researchers. Several studies have been published lately on the effectiveness of the PlyoML approach in the rehabilitation of children with ULCP [15,16,22,23,24,25]. In aggregate, the evidence derived from these studies establishes that PlyoML intervention lasting eight or twelve weeks is worthwhile for enhancing muscle strength, balance, postural control, weight-bearing symmetry, and gait characteristics.

Both training models (i.e., PsCS and PlyoML) are associated with a number of neuromuscular adaptations. The PsCS relies on the postulate of trunk stability, develops strength and endurance, particularly in the core muscles, and enhances mobility, efficiency, and movement control [26]. On the other side, PlyoML enhances power and explosiveness, strength and endurance, neuromuscular coordination, and proprioception, especially in the lower body [21,27]. In consideration of the foregoing, we thought that children with ULCP would most likely take advantage of these underlying adaptations if both training approaches were combined. Accordingly, this study was designed to compare the effects of PsCS, PlyoML, and their combination on postural control, balance, and mobility performance in children with ULCP. We hypothesized, based on previous evidence [13,14,15,16,22,23,24], that combined PsCS and PlyoML would induce greater improvements in all outcome measures compared to unimodal PsCS and PlyoML.

## 2. Materials and Methods

### 2.1. Design and Ethics

A prospective, assessor-blinded, randomized study design with an intention-to-treat analysis was adopted. The study was undertaken between February 2021 and April 2022 at the university’s Biomechanics/Balance Laboratories and Physical Rehabilitation Center, Al-Kharj, KSA. The protocol was approved by the Physical Therapy Research Ethics Committee on 24 January 2021 (Authorization No: RHPT/0021/0114). The methodologies used in this experiment followed the ethical standards set forth in the updated version of the Declaration of Helsinki [28]. To ensure methodological rigor and transparent reporting, this study was meticulously conducted in accordance with the Consolidated Standards of Reporting Trials (CONSORT) guidelines. The CONSORT statement served as a comprehensive framework, guiding the entire research process, including study design, data collection, and result dissemination. Participants and their families were informed about all aspects of the study (i.e., objectives, experimental procedures, benefits, and potential risks) before enrollment, and were required to sign a consent form when they decided to participate. The study protocol was retrospectively registered at ClinicalTrial.gov (Identifier: NCT05429281).

### 2.2. Participants

Eighty-one children were recruited from Pediatric Neurology/Physical Therapy clinics of three referral hospitals and the university’s Physical Rehabilitation Center at Al-Kharj, KSA. Inclusion criteria were (i) a child’s diagnosis of spastic ULCP confirmed by a neuro-pediatrician [29], (ii) gross motor function classification system (GMFCS) level I–II [30], (iii) age between 12 and 18 years, (iv) mild spasticity grade 1 or 1+ per the Modified Ashworth Scale [31], and (v) ability to understand multi-step instructions. Exclusion criteria were (i) neurolytic blocking agents within the past six months, (ii) corrective orthopedic/neuromotor surgeries within the past year, (iii) sensory–perceptual impairments, (iv) cardio-respiratory disorders that preclude safe engagement in programmed interventions, and (v) inability to attend required intervention/measurement sessions.

#### 2.2.1. Sample Size Determination

To detect a decisive difference between study groups, we conducted a priori power analysis utilizing PASS software, v16.0.12 (NCSS, Kaysville, UT, USA). The analysis was based on data collected from a pilot study, using the primary outcome measure [i.e., the overall dynamic limits of stability (LoS) data, representative of postural control]. In a three-group ANOVA study, a sample size of 63 children (i.e., 21 children per group) was required to achieve 92% power to detect a minimum difference of 6.24 in the overall LoS using the multiple pairwise comparison (Tukey–Kramer) test, at an alpha level of 0.05, assuming a common within-group standard deviation of 3.61. However, we enrolled up to 81 children (27 for each group) in anticipation of a withdrawal rate of approximately 20%. 

#### 2.2.2. Randomization Procedure

Children were randomized to the PsCS, PlyoML, or combined group by an independent researcher who was not involved in the current study. To create a balance of potential clinical/prognostic factors among the study groups, a stratified permuted block randomization procedure was carried out. First, stratification by age and GMFCS level was performed during the selection phase. Then, permuted block randomization for each stratum was accomplished through an online randomization tool (www.randomization.com; accessed on 31 December 2020). The randomization was done in blocks of varying sizes (with a maximum of nine and a minimum of three in each block), thereby ensuring equal numbers were allocated to each group. In each block, a sequence of numbered, closed, non-transparent envelopes was constructed. The researcher unlocked the next envelope in the sequence after each child had formally entered the study.

### 2.3. Outcome Measures

The postural control, balance, and mobility variables were assessed on pre- and post-intervention occasions. Measurements were carried out by two trained outcome assessors, with one assessor responsible for postural control assessment and the other for balance and mobility assessment. Both assessors underwent rigorous training to standardize the assessment procedures. The assessors were blinded to the intervention assigned to each participant and did not get involved in the delivery of the intervention to ensure unbiased ascertainment of outcomes. Prior to the data-collecting session, each participant attended an orientation session to learn about the assessment processes and how to properly carry out the required assessments. All measurements were performed in one session in the morning (between 9 and 12 am) and started with a postural control test, followed by balance and mobility tests (CB&M, FWT, then TUDS). A 10 min rest period was allowed in between. 

#### 2.3.1. Postural Control

Postural control was evaluated with the Biodex balance system (BBS; Biodex Medical System, Shirley, NY, USA). During the LoS test, participants were challenged to displace and control their center of gravity (CoG) within their base of support (BoS) while standing. Directional LoS (the peak CoG excursions participants were able to purposefully cover in the backward, forward, paretic, and non-paretic directions) as well as overall LoS (the mean of all directions) were assessed with eyes open on a fully unstable balance platform (i.e., the platform stability was set at level 1, the most unstable stable level among 12 stability levels). 

Participants had to stand barefooted on the balance platform to eliminate any potential interference or alteration in the sensory input from the feet or mechanics of balance, enabling a more accurate assessment that reflected the individuals’ inherent postural control abilities, and were instructed not to utilize the handrails during the assessment. They were required to shift their body weight to move a cursor (representing the CoG) from a central target (indicating the center of pressure) so as to intercept a blinking target before going back again to the central position, as quickly and with as little deviation as they could, keeping their body straight, and using their ankles as primary axes of movement. The same process was followed to complete eight targets (arranged at intervals of 45^o^ around the central target and 50% of the maximum LoS), which protruded on the display panel in random order. This process involves the transformation of the angular motion of the lean into linear movement of the CoG, represented on the display panel. The LoS test measured the accuracy (%) with which participants transferred their CoG to intercept the targets, based on 100% being a straight line. Three trials were permitted for each participant and average directional and overall LoS values were employed for the subsequent analyses. Higher values indicate greater postural control [6,32].

#### 2.3.2. Balance and Mobility

Balance and functional mobility were assessed through three outcome measures: the Community Balance and Mobility Scale (CB&M) [33], the Functional Walking Test (FWT) [34], and the Timed Up and Down Stair test (TUDS) [35]. 

The CB&M is a valid, performance-based, and clinically meaningful measure appraising high-level balance capabilities while also addressing the speed and coordination components necessary for ordinary community function in children/adolescents with neurological disorders who, although ambulatory, have balance impairments affecting their engagement in the community [33]. The CB&M consists of 13 challenging tasks, six of which are completed on both paretic and non-paretic sides (i.e., totaling 19 items). Each item is scored on a six-point rating scale (i.e., a 0–5 scale reflecting the progressive difficulty of the task) that uses precise response option descriptors. A score of 0 indicates “complete inability to perform the task”, and a score of 5 denotes “the most successful completion of the item possible”. A bonus (+1) is possible for the 12th item, allowing for a maximum score of 96. For each item, scoring is completed using the first trial. All tasks were completed without ambulation aids, although orthotics were allowed to be worn. To ensure adequate understanding, the assessor gave the participants verbal instructions and demonstrated all of CB&M’s items. 

The FWT is an easy-to-administer, reliable, and valid measure developed to specifically reflect balance related to walking and to assess functional walking capacity in ambulant children/adolescents with ULCP (within the activity level of the International Classification of Functioning model of disability) aged between 4 to 18 years [34]. The FWT is an ordinal scale score made up of 11 items, broken into five categories. The test items include kneel walking, transitions to standing, incline walking, ascending/descending stairs, and walking a narrow beam, all of which focus on the postural control and balance components of walking. The participants were assessed while wearing their customary footwear and orthosis. The FWT has a maximum score of 23, with a greater score reflecting better functional walking capacity. 

The TUDS test is a quick, low-cost, and reliable measure for assessing functional mobility in ambulant children/adolescents with CP who are functioning at GMFCS level I or II [35]. Participants were required to stand up 30 cm away from the bottom of a 14-step stair flight (each step was 20 cm in height and 36.5 cm in depth), ascend as quickly as they felt safe and comfortable to the top, turn around, and descend to the starting point. The test was performed with the participants putting on their usual footwear. Stair climbing strategies included, but were not limited to, running up the stairs, skipping steps, and foot-over-foot/step-to sequence. Verbal cues like “ready”, “set”, and “go” were given. The test was scored as the time (in seconds) to complete the task by using a handheld stopwatch (Sportline Sport Timer; Sportline, Yonkers, NY, USA), and each data point reflects the time for one trial. Lower times in the test reflect better functional mobility.

### 2.4. Interventions

#### 2.4.1. Pilates-Based Core Strengthening

The PsCS exercises were executed over ~45 min/session, for a total of 24 sessions spread over a course of 12 weeks (i.e., two sessions per week). The program was designed to translate PsCS principles, which include recruiting the deep stabilizing muscles of the body center to prepare movement, maintaining a neutral pelvis and shoulder girdle to allow the extremities to dissociate from the trunk, and paying attention to every aspect of each exercise to achieve correct movements. The training was directed by two pediatric physical therapists trained in PsCS (i.e., experience >5 years). The fundamentals of PsCS were thoroughly discussed with the participants in the first session and were repeatedly mentioned at the beginning of each session. The exercise protocol maintained a high degree of standardization and consistency across all participants. Specifically, participants engaged in level I and II mat workouts, which were generally adapted to meet the physical demands associated with the specific age group under investigation and the constraints imposed by ULCP. The training followed a carefully designed progression that increased in difficulty within each session. Initially, participants were instructed to engage their deep stabilizing muscles while performing exercises that presented a modest level of challenge. These exercises involved making small, controlled movements to activate and strengthen the targeted muscles. As participants progressed, they were gradually introduced to more demanding positions that required a greater amount of effort from the deep stabilizers and simultaneous engagement of multiple muscle groups. In this phase, participants were encouraged to perform larger, controlled movements, further challenging their strength, stability, and control [36]. The same activities were kept up, but more repetitions were added on a 4-week basis (i.e., in three blocks). Each session commenced with a warm-up, which was achieved through centering and segmental limb movements in the supine position and ended with a cool-down in the form of stretching, postural, and relaxation exercises. Where necessary, individualized facilitation strategies (visual/mental imagery, verbal/tactile cueing, or presentation) were provided by the therapist to control speed, correct the technique, activate appropriate muscles, or modify the exercise to suit the needs of the participants. Specifics of the PsCS program are outlined in Supplementary Table S1.

#### 2.4.2. Plyometric-Based Muscle Loading

The PlyoML exercises were performed for 45 min/session, twice/week (interspersed with 2-day recovery periods), over 12 successive weeks under the surveillance of two licensed pediatric physical therapists who possessed extensive expertise (for more than five years) in delivering plyometrics. All participants were taught how to correctly execute the PlyoML exercises during a pre-intervention session. The training was geared predominantly toward the lower body and was carried out in consideration of the safety/performance guidelines set forth by the US National Strength and Conditioning Association [37]. The program was carried out as previously reported [15], and included 10 exercises, with a variety of bilateral and unilateral hopping, jumping, or bounding activities, orientated in the horizontal and vertical planes. Training progression was achieved by increasing the number of sets and/or repetitions in three 4-week blocks. The training began with 65 foot contacts each session in the first block, increased to 165 foot contacts per session in the second block, and ended up with 195 foot contacts per session in the third block. A full description of exercises and their progression is outlined in Supplementary Table S2. The PlyoML workout was preceded by a 5 min warm-up and ended up with a 5 min cool-down. Participants were stimulated to maximize their efforts as much as possible during the execution of each exercise (e.g., horizontal distance; vertical height; reduced ground contact time). All repetitions in a given set were completed continuously (i.e., no inter-repetition rest), with 1–2 min of inter-exercises. All workouts were done on a rubber surface while participants wore cushioned footwear.

#### 2.4.3. Combined Intervention

To match groups for total training repetitions, participants in the combined intervention group performed a combination of PsCS and PlyoML training, but with a 50% number of sets/repetitions of each exercise. The training was repeated two times a week, totaling 24 sessions over 12 successive weeks. PsCS and PlyoML were executed within the same session, with a 10–15 min rest interval, to ease the burden on participants and their families from attending frequently to the rehabilitation center. In each session, the PsCS training was completed before moving on to PlyoML.

### 2.5. Data Analysis

Statistical analyses were accomplished through the Statistica software, version 12 (Statsoft, TIBCO Software, Palo Alto, CA, USA). Unless otherwise stated, variables were expressed as mean ± standard deviation. The Shapiro–Wilk test was employed to check departures from the Gaussian distribution. Where appropriate, a logarithmic transformation was undertaken. The two-way mixed-model ANOVA test [one repeated-measures factor, time (pre/post), and one between-subject factor, group (PsCS/PlyoML/combined intervention)] was used to compute the pre-to-post change differences among the study groups. These differences were decided by the group-by-time interaction effect. Tukey’s post-hoc test was used, in cases where the interaction effect was significant, to compare the study groups on a pair-by-pair basis. The dependent sample *t*-test was used, whenever the null hypothesis for mixed ANOVA was rejected, to estimate changes within each group. The partial eta-squared (*η*^2^_Partial_) and Hedges’ g formulae were used to find out the size of the significant between-group and within-subject effects, respectively. The null hypothesis was rejected at a *p*-value of <0.05 across all statistical tests. 

## 3. Results

**Figure 1:**
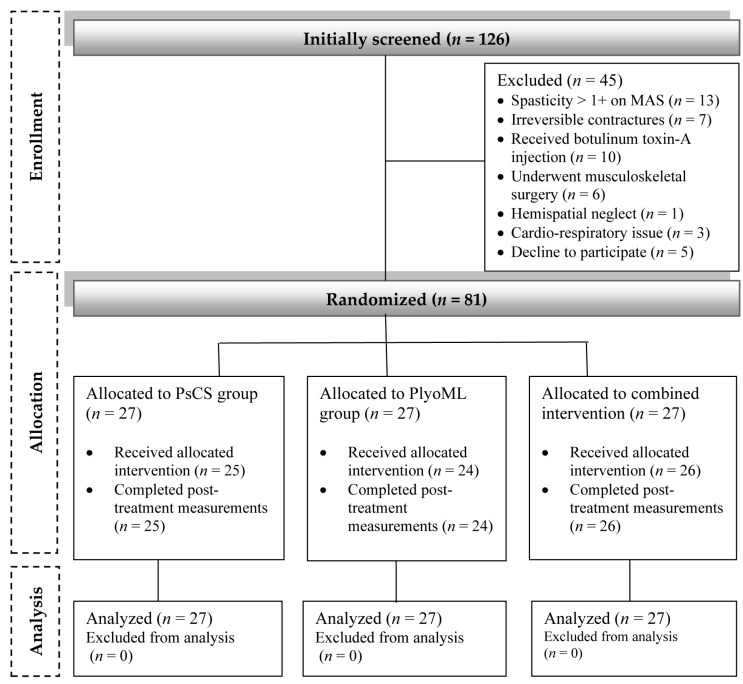
A comprehensive flowchart illustrating the participants’ flow and retention through the study phases.

A comprehensive flowchart illustrating the participants’ flow and retention through the study phases is presented in Figure 1. Eighty-one children (out of 126 potentially eligible children) fulfilled the inclusion criteria and were randomly allocated into the study groups. Six children (~8.6%; two from the PsCS group; three from the PlyoML group; and one from the combined-intervention group) either did not complete the allocated intervention or missed the follow-up measurements. The reasons for withdrawal/loss were traveling outside the working area, scheduling conflicts, or undisclosed personal issues. While the intention-to-treat principle was adopted for this study, their missing data were substituted through a multiple-imputation approach and were incorporated into the analysis. The baseline demographic and clinical characteristics are summarized in Table 1. No differences were observed at baseline (pre-intervention) between the PsCS, PlyoML, and combined-intervention groups concerning age, sex distribution, or anthropometric properties (*p* > 0.05). Besides, the three groups were comparable in terms of their clinical attributes (i.e., paretic side, spasticity grade, and GMFCS level) (*p* > 0.05).

The pre-to-post-change differences in postural control among the study groups are summarized in Table 2. The two-way ANOVA analysis revealed a significant intervention-by-time interaction effect on directional LoS [backward (*F*_2,78_ = 6.45, *p* = 0.003, *η*^2^_Partial_ = 0.14), forward (*F*_2,78_ = 11.40, *p* < 0.001, *η*^2^_Partial_ = 0.23), paretic limb (*F*_2,78_ = 5.83, *p* = 0.004, *η*^2^_Partial_ = 0.13), and non-paretic limb (*F*_2,78_ = 3.89, *p* = 0.024, *η*^2^_Partial_ = 0.09)] as well as on the overall LoS (*F*_2,78_ = 16.69, *p* < 0.001, *η*^2^_Partial_ = 0.29). The Tukey post-hoc test showed a larger increase in directional LoS in the combined intervention group compared to either the PsCS group [backward (*p* = 0.006), forward (*p* = 0.015), paretic limb (*p* = 0.017), and non-paretic limb (*p* = 0.006)] or the PlyoML group [backward (*p* = 0.033), forward (*p* = 0.036), paretic (*p* = 0.018), and non-paretic (*p* = 0.004) directions], and this was also the case for overall LoS (*p* < 0.001).

The pre-to-post-change differences in balance and mobility between the study groups are depicted in Table 3. The analysis detected a significant intervention-by-time interaction effect on the CB&M (*F*_2,78_ = 12.82, *p* < 0.001, *η*^2^_Partial_ = 0.25), FWT (*F*_2,78_ = 10.23, *p* < 0.001, *η*^2^_Partial_ = 0.21), and TUDS (*F*_2,78_ = 17.10, *p* < 0.001, *η*^2^_Partial_ = 0.31). The post-hoc pairwise analysis indicated greater increases in CB&M and FWT scores in the combined intervention group when compared to either the PsCS (*p* = 0.037 and *p* = 0.002, respectively) or PlyoML (*p* = 0.012 and *p* = 0.038, respectively). Also, there was a significant reduction in TUDS duration in the combined intervention group relative to both the PsCS (*p* = 0.046) and the PlyoML (*p* = 0.021).

## 4. Discussion

The present study endeavored to compare the effects of PsCS, PlyoML, and their combination on postural control, balance, and mobility performance in children with ULCP. Our novel findings suggest that, compared to unimodal PsCS or PlyoML, their combination induces greater enhancements in postural control in children with ULCP, as evidenced by their greater capacity to move/control the CoG within the BoS during the dynamic LoS test. Additionally, the combined PsCS and PlyoML training regimen leads to greater improvements in balance and mobility functions, as reflected by the better performance on the CB&M scale, FWT, and TUDS test. Current novel findings may have potential clinical and practical implications, which are highlighted in the following paragraphs.

The consequences of single-mode PsCS training have been explored in several studies involving children with ULCP. In a recent investigation by Adıguzel and Elbasan [13], PsCS training (carried out twice/week in an 8-week program), in comparison with conventional NDT, resulted in more favorable changes in postural control, balance, and gait performance in children with ULCP. Also, a newly released study by Abd-Elfattah et al. [14] found that PsCS training (45 min/session, thrice/week for 10 weeks) was a helpful adjunct to the usual physical therapy for enhancing balance capability and gross motor functions in children with spastic diparetic CP. Further, a single-case study by Dos Santos et al. [17], revealed that after reformer PsCS training for eight weeks for a child with ULCP functioning at GMFCS level I, lower limb strength improved bilaterally, with more gain on the paretic side, and the child’s capacity for controlling his posture increased as the anterior–posterior and medial–lateral displacement of the body’s center of pressure in a static stance decreased. Therefore, data from the PsCS group in the present study seems to be consistent with the aforementioned reports, adding to the growing body of evidence that such a training paradigm has positive implications for several components of the motor abilities of children with ULCP. The PsCS, being a “mind–body” exercise, may have contributed to teaching children to control their bodies, especially since such an exercise paradigm emphasizes cognitive concentration to move the body at a slow, deliberate, and flowing pace [18]. The current PsCS was focused on building strength in the core muscles (i.e., abdominal, back, and pelvic floor muscles), engaging these muscles through a series of controlled movements and breathing techniques. By strengthening the core muscles, PsCS could have improved posture, balance, and axial stability [38]. The PsCS might have further promoted motor coordination, body awareness, flexibility, strength, and endurance, all of which are essential for efficient postural control and functional performance [26,39].

Evidence on the role of independent PlyoML training for children with ULCP has also been characterized in multiple investigations. Elnaggar and colleagues [15,16,22,23,24,25] recently conducted a series of randomized controlled trials exploring the emerging role of PlyoML in children with ULCP. They established that an 8–12-week PlyoML program (45–60 min/session, repeated two or three times a week) is an effective training strategy for improving lower limb muscle strength and activation pattern [15,23,24], increasing weight shift toward the paretic side [23], enhancing postural control and balance capability [15,16,22,24], rectifying spatiotemporal gait asymmetries [16], and boosting functional and locomotor capacity [22,25]. Johnson et al. [40], conducted a multiple-baseline, multiple-probe, single-subject experiment to evaluate the effects of PlyoML on gross motor abilities in three children with ULCP. Their results indicated significant improvements in muscle strength and gross motor function after PlyoML training for 8–14 weeks. Hence, the findings of the present study (PlyoML group) support in part the work of previous studies in this area linking PlyoML training with improved postural control, balance, and mobility in children with ULCP. Indeed, the beneficial effects of PlyoML on balance performance were recently confirmed in a meta-analysis [41]. Considering the explosive and dynamic nature of PlyoML exercises, they may have promoted some neuromotor adaptations such as heightened muscle reflexes, re-established motor activation patterns, and enhanced proprioceptive consciousness, resulting in increased neuromotor efficiency, meaning that the nervous system learned to engage the appropriate muscles, regulate muscular activities, and stabilize the body segments during dynamic constraints [15,25,42]. The PlyoML training provides a broad array of balance challenges because the CoG moves continually in the vertical and horizontal planes during exercise performance, through which the children’s ability to maintain balance and posture increased [16].

As discussed previously, both PsCS and PlyoML interventions probably induced physiological–biomechanical adaptations related to the specific exercises of each training mode, explaining the improvements in the PsCS and PlyoML groups. However, the greater improvements in the combined PsCS–PlyoML group suggest combined physiological benefits (i.e., no interference effect) [43]. Alternatively, the PsCS, performed ahead of the PlyoML in the present study, may have favored a pre-conditioning effect during the PlyoML session [44]. The PsCS has been shown to reduce body sway during static standing and enhance core stability through the stiffening of trunk musculatures [17,38], representing essential factors in preparation for the lower limb movements during the PlyoML and for efficient performance so as to enhance training outcomes. However, future studies may analyze the optimal within-session distribution of PlyoML and PsCS exercises.

Of note, our study matched total repetitions in the three intervention groups, a key methodological aspect of exercise dosing [45], Additionally, the sample size in each group allowed adequate statistical power, a methodological issue that offers more robust results compared to studies with reduced samples, a limitation quite common in exercise science literature, including core-based and plyometric-based interventions [46,47,48]. Further, our study employed three different clinical measures of balance and mobility, which enabled us to evaluate the intervention effects in different functional contexts. Furthermore, the 12-week duration of the training was long enough to notice significant changes in functional performance.

The aforementioned methodological aspects of our study increase the probability of drawing a solid conclusion concerning the comparability of the combined versus unimodal training regimens. However, while interpreting the findings of this study, certain limitations should be taken into account. First, our sample specifically targeted children with ULCP (functioning at GMFCS level I or II), who exhibited milder disabilities in comparison to children with other types of CP or more severe subtypes of ULCP. Moreover, the current evidence is derived from data collected within a specific age range (i.e., 12–18 years). Consequently, it remains uncertain whether the findings presented in this study can be broadly extrapolated to encompass children with CP who function at different GMFCS levels or belong to a younger age group. Second, muscle strength, a crucial indicator of Pilates/plyometric qualities, and patient/family perceptions of interventions, essential measures that provide valuable insights into their perspectives and preferences and capture multidimensional aspects of health and well-being that may not be adequately tracked by clinical measures alone, were not evaluated in the present study. For a clearer and more holistic understanding of intervention effects, additional investigations that take both clinical and patient/family-centered assessments into account will, therefore, need to be undertaken. Third, the current study did not assess changes in proprioception or muscle activation patterns, all of which are important determinants of postural control, balance, and mobility. Therefore, additional studies are needed to assess the physiological and biomechanical factors underlying the improvements in postural control, balance, and mobility in children with ULCP after combined PsCS and PlyoML exercise programs. Fourth, the lack of established Minimal Clinically Important Difference (MCID) values for the outcome measures employed herein is an important limitation. The MCID signifies the smallest change in a measure that is considered clinically meaningful and relevant to patients. The lack of established MCID values poses a challenge in determining the practical significance of the observed differences. It is, therefore, crucial for future research to prioritize the establishment of MCID values for the outcome measures employed. This endeavor would provide a more comprehensive understanding of the clinical impact of the treatments and enhance the interpretation of the study findings. Fifth, the current finding only demonstrates the short-term, post-treatment effects. Accordingly, caution must be applied, as the sustainability of the intervention effects remains questionable. If the debate is to be moved forward, further studies on this question would help determine the long-term repercussions. Finally, the present study lacks a control (no-training) group. Forthcoming studies should thus think about integrating control participants (without exposure to training) to confirm that results are truly produced by the intervention rather than chance.

## 5. Conclusions

In summary, the present study contributes substantial evidence supporting the superiority of a combined PsCS and PlyoML exercise program over independent PsCS or PlyoML interventions in augmenting postural control, balance, and mobility among children with ULCP. The implications of this research extend to physical rehabilitation practitioners, as it informs their decision-making process regarding the choice between integrated and independent interventions and is grounded in reliable empirical knowledge. There is, however, a need for future exploratory investigations elucidating the underlying mechanisms through which these exercises yield improvements in the outcome measures. Such studies could subsequently propose strategies to further optimize exercise prescriptions for this patient population.

## Figures and Tables

**Table 1:** Baseline demographic and clinical characteristics of the participating children.

	PsCS Group (*n* = 27)	PlyoML Group (*n* = 27)	Combined Group (*n* = 27)	*p*- Value
Age, year	15.19 ± 1.84	14.78 ± 1.45	15.56 ± 1.63	0.23 ^‡^
Gender (b/g), *n* (%)	18 (66.7)/9 (33.3)	16 (59.3)/11 (40.7)	20 (74.1)/7 (25.9)	0.56 ^§^
Side affected (RT/LT), *n* (%)	8 (29.6)/19 (70.4)	6 (22.2)/21 (77.8)	4 (14.8)/23 (85.2)	0.48 ^§^
MAS level (1/1+), *n* (%)	14 (51.9)/13 (48.1)	12 (44.4)/15 (55.6)	16 (59.3)/11 (40.7)	0.59 ^§^
GMFCS level (I/II), *n (%)*	15 (55.6)/12 (44.4)	17 (63)/10 (37)	19 (70.4)/8 (29.6)	0.58 ^§^
Height, cm	155.6 ± 11.1	154.7 ± 9.3	156.6 ± 10.3	0.79 ^‡^
Weight, Kg	52.30 ± 7.58	51.81 ± 6.43	54.37 ± 7.29	0.38 ^‡^
Body mass index, Kg/m^2^	21.51 ± 1.41	21.59 ± 1.24	22.11 ± 1.45	0.22 ^‡^

Categorical data are expressed as frequency (%), and numerical data are shown as mean ± standard deviation. Abbreviations: PsCS: Pilates-based core strengthening, PlyoML: plyometric-based muscle loading, b/g: boy/girl, RT: right, LT: left, GMFCS: gross motor function classification system, MAS: modified Ashworth scale. *p* values: ^‡^ 1-way ANOVA test, ^§^ Pearson’s χ^2^ test.

**Table 2:** Changes in postural control variables between and within the study groups.

	PsCS Group (*n* = 27)	PlyoML Group (*n* = 27)	Combined Group (*n* = 27)	Interaction Effect	
				*p*-Value	*η^2^*_Partial_
Directional LoS—backward					
Pre	39.92 ± 4.47	40.52 ± 5.51	42.10 ± 4.99	0.003 *	0.14
Post	42.74 ± 6.52	43.81 ± 4.92	49.15 ± 6.55		
*p*-value	0.004 *	0.002 *	<0.001 *		
Hedges’ g (95% CI)	0.49 (0.14–0.86)	0.61 (0.22–1.03)	1.18 (0.76–1.65)		
Directional LoS—forward					
Pre	42.52 ± 4.47	42.81 ± 4.45	43.33 ± 6.95	<0.001 *	0.23
Post	44.48 ± 6.15	45.11 ± 5.75	51.92 ± 7.81		
*p*-value	0.014 *	0.007 *	<0.001 *		
Hedges’ *g* (95% CI)	0.35 (0.07–0.65)	0.43 (0.12–0.77)	1.13 (0.63–1.68)		
Directional LoS—paretic					
Pre	45.41 ± 8.35	45.15 ± 9.09	48.52 ± 11.19	0.004 *	0.13
Post	48.26 ± 7.10	48.56 ± 9.12	57.59 ± 8.50		
*p*-value	0.023 *	0.0006 *	0.0001 *		
Hedges’ *g* (95% CI)	0.36 (0.05–0.68)	0.36 (0.16–0.59)	0.89 (0.44–1.37)		
Directional LoS—non-paretic					
Pre	53.37 ± 5.76	52.48 ± 5.34	54.93 ± 6.44	0.024 *	0.09
Post	56.67 ± 7.64	57.63 ± 6.48	63.11 ± 8.45		
*p*-value	0.006 *	0.0005 *	<0.001 *		
Hedges’ *g* (95% CI)	0.47 (0.14–0.83)	0.84 (0.37–1.35)	1.06 (0.63–1.54)		
Overall LoS					
Pre	45.31 ± 2.80	45.24 ± 3.39	47.21 ± 4.55	<0.001 *	0.29
Post	48.04 ± 3.96	48.78 ± 3.27	55.44 ± 4.39		
*p*-value	0.0004 *	<0.001 *	<0.001 *		
Hedges’ *g* (95% CI)	0.77 (0.35–1.23)	1.04 (0.61–1.50)	1.79 (1.20–2.47)		

Pre- and post-intervention data are shown as mean ± standard deviation. *η*^2^_Partial_: ANOVA effect size, Hedges’ *g*: *t*-test effect size. * significant at *p* < 0.05. Abbreviations: PsCS: Pilates-based core strengthening, PlyoML: plyometric-based muscle loading, LoS: limits of stability, CI: confidence interval.

**Table 3:** Changes in balance and mobility variables between and within the study groups.

	PsCS Group (*n* = 27)	PlyoML Group (*n* = 27)	Combined Group (*n* = 27)	Interaction Effect	
				*p*-Value	*η^2^*_Partial_
CB&M, /96					
Pre	62.35 ± 6.78	59.21 ± 5.79	61.22 ± 7.60	<0.001 *	0.25
Post	64.94 ± 5.47	66.73 ± 6.18	74.01 ± 8.91		
*p*-value	0.022 *	<0.001 *	<0.001 *		
Hedges’ *g* (95% CI)	0.41 (0.06–0.77)	1.22 (0.74–1.76)	1.50 (0.92–2.15)		
FWT, /23					
Pre	16.88 ± 2.37	17.10 ± 2.25	17.27 ± 2.33	<0.001 *	0.21
Post	17.49 ± 2.03	18.29 ± 1.90	20.57 ± 1.96		
*p*-value	0.005 *	0.032 *	<0.001 *		
Hedges’ *g* (95% CI)	0.27 (0.08–0.47)	0.55 (0.04–1.10)	1.49 (0.91–2.14)		
TUDS, seconds					
Pre	18.31 ± 4.10	19.10 ± 4.43	18.36 ± 4.31	<0.001 *	0.31
Post	16.17 ± 2.70	15.96 ± 3.14	11.78 ± 2.34		
*p*-value	0.002 *	<0.001 *	<0.001 *		
Hedges’ *g* (95% CI)	0.59 (0.22–0.99)	0.79 (0.45–1.18)	1.84 (1.31–2.48)		

Pre- and post-intervention data are shown as mean ± standard deviation. *η*^2^_Partial_: ANOVA effect size, Hedges’ *g*: *t*-test effect size. * significant at *p* < 0.05. Abbreviations: PsCS: Pilates-based core strengthening, PlyoML: plyometric-based muscle loading, LoS: limits of stability, CI: confidence interval.

## Data Availability

The data that support the findings of this study are available on reasonable request from the corresponding author due to privacy.

## Supplementary Materials

The following supporting information can be downloaded at https://www.mdpi.com/article/10.3390/children11020243/s1, Table S1: The Pilates-based core strengthening program, performance instructions, and training progression; Table S2: Plyometric-based muscle loading exercises, performance instructions, and training progression.
