# Supplementary material for: Optimization of Postural Control, Balance, and Mobility in Children with Cerebral Palsy: A Randomized Comparative Analysis of Independent and Integrated Effects of Pilates and Plyometrics
Source: Children (Basel). 2024 Feb 15;11(2):243. doi: 10.3390/children11020243 (PMC10887404; doi:10.3390/children11020243)
Supplement: Supplementary file 1 [file children-11-00243-s001.zip › Supplementary Table S1.docx]

**Supplementary Table S1:** The Pilates-based core strengthening program, performance instructions, and training progression.

| Exercise | Performance instructions | Wk. 1-4 | Wk. 5-8 | Wk. 9-12 |
| --- | --- | --- | --- | --- |
| Prone bridge | - Lay prone, with elbows under shoulders. - Start with breathing in prone. - Lift the hip, knees, and pelvis off the floor during expiration. - Keep the head, shoulders, pelvis, knees, and ankle in a straight line for 10 sec. | 2 sets x 4 reps. | 2 sets x 6 reps. | 2 sets x 8 reps. |
| Shoulder bridge | - Lay down on the back, hands by sides, knee bent, and feet flat on the floor. - Raise hips slowly to create a straight line from hips to knees during inspiration. - Hold that position for 5-10 sec. - Lower hips slowly back to the floor during expiration. | 2 sets x 4 reps. | 2 sets x 6 reps. | 2 sets x 8 reps. |
| Unilateral shoulder bridge | - Lay down on the back, hands by sides, keep the knee of one leg bent and foot flat on the floor, and the other leg straight off the floor. - Lift the pelvis up to the point where the legs and torso are essentially in a straight line during inspiration. - Hold that position for 5-10 sec. - Slow and control on the way down to the floor during expiration. - Repeat with exchanging the position of both legs. | 2 sets x 4 reps. (RT)  2 sets x 4 reps. (LT) | 2 sets x 6 reps. (RT)  2 sets x 6 reps. (LT) | 2 sets x 8 reps. (RT)  2 sets x 8 reps. (LT) |
| Side bridge | - Lie on one side with the forearm on that side supporting on the floor and your hand on the other side on your waist. NB: The feet were stabilized by the therapist. - Raise your hips to straighten your body, and hold for 5 sec. - Drop your hips down without touching the floor, then lift them again - Repeat on the other side. | 1 set x 4 reps. (RT)  1 sets x 4 reps. (LT) | 1 set x 6 reps. (RT)  1 sets x 6 reps. (LT) | 1 set x 8 reps. (RT)  1 set x 8 reps. (LT) |
| Hundreds | - Lie on your back with your knee bent up toward the ceiling, feet flat on the floors, and arms by your sides. - Start taking in a breath, and move your chin toward your chest so that your eyes looking at your knees. - Lift your head and shoulders off the floor as you breathe out, and raise your arms so your hands are at the level of your hips. - While holding this position take in a breath to the count of 5 while bouncing arms up and down and keeping other parts of your body still. - Slowly rest the head, shoulders, and arms back to the floor. | 2 sets x 2 reps. | 2 sets x 4 reps. | 2 sets x 6 reps. |
| Leg pull supine | - Sit with both legs straight out in front, holding them together. - Extend your arms behind your body, palms down, fingers pointing forward. - Breath out, and lift the pelvis off the floor to the point where the head, shoulders, pelvis, knees, and ankles are essentially in a straight line. - Take in a breath, then, hinge in the hip joint and lower the pelvis down. | 2 sets x 6 reps. | 2 sets x 8 reps. | 2 sets x 10 reps. |
| Mermaid side stretch | - Sit on the floor with both legs folded to the right side such that the front foot is placed on the opposite inner thigh and the back foot flat to the floor, keeping the right hand settled onto the floor. - Extend your left arm straight up and above your head and in front of the ear. - Keeping the left pelvis on the floor, reach left arm up towards the ceiling and over to the right side to the extent that nowhere left to go with the stretch, by letting the right elbow bend to the floor. - Deepen the left stretch by sending the left hip down, hold, and take a few breaths. - Return to upright sitting by sending the left pelvis down to the mat and using abdominal muscles to bring the torso back. - Reverse the stretch to the other side | 2 sets x 4 reps. (RT)  2 sets x 4 reps. (LT) | 2 sets x 6 reps. (RT)  2 sets x 6 reps. (LT) | 2 sets x 8 reps. (RT)  2 sets x 8 reps. (LT) |
| Side laying clam | - Lie on one side with hips and feet stacked, and knees bent 90^o^. and head resting on the arm as it is bent or stretched overhead. - Draw knees in toward the body till feet come in line with the pelvis. - Take in a breath, raise the top knee as far as feasible keeping feet together, without disturbing the alignment of the hips. - bring your knee slowly back to the starting position while breathing out. - Repeat on the other side. | 2 sets x 4 reps. (RT)  2 sets x 4 reps. (LT) | 2 sets x 6 reps. (RT)  2 sets x 6 reps. (LT) | 2 sets x 8 reps. (RT)  2 sets x 8 reps. (LT) |
| - Each session started with a 5-minute warm-up (centering and segmental limb movements) and ended with a 5- min cooldown (trunk rotation exercises from supine, and knee to chest exercises performed unilaterally, then bilaterally). - Children were given rest intervals as needed and also when they reported any pain and/or discomfort during the training. - Abbreviations: reps: repetitions, RT: right side, LT: left side, sec: second | | | | |
