# Supplementary material for: Optimization of Postural Control, Balance, and Mobility in Children with Cerebral Palsy: A Randomized Comparative Analysis of Independent and Integrated Effects of Pilates and Plyometrics
Source: Children (Basel). 2024 Feb 15;11(2):243. doi: 10.3390/children11020243 (PMC10887404; doi:10.3390/children11020243)
Supplement: Supplementary file 1 [file children-11-00243-s001.zip › Supplementary Table S2.docx]

**Supplementary Table S2:** Plyometric-based muscle loading exercises, performance instructions, and training progression.

| Exercise | Characterization | Block 1 | Block 2 | Block 3 |
| --- | --- | --- | --- | --- |
| Horizontal plyometric exercise-model | | | | |
| Bound | Push off one foot and jump out and forward as far as possible to land on the other foot. | 1 x 5 | 1 x10 | 1 x15 |
| Forward-jump | With a forward arm swing, jump as far as possible with both feet. | 1 x 5 | 1 x10 | 1 x15 |
| Single-leg forward hop | Hop forward between lines with no stops in both directions. | 1 (5RT and 5LT) x 5 | 1 (5RT and 5LT) x 10 | 1 (5RT and 5LT) x 15 |
| Lateral leap | Stand on one leg, stretch out the other leg, then hop laterally. | 1 x 5 | 1 x10 | 1 x15 |
| Side-to-side jump | Jump as far as possible with both feet from one side to the other. | 1 x 5 | 1 x10 | 1 x15 |
| Vertical plyometric exercise-model | | | | |
| Reciprocal stride-jump | From stride standing, jump up, with feet interchangeably advanced forward between jumps. | 1 x 5 | 1 x10 | 1 x15 |
| Squat-jump | Stand with feet shoulder-width apart and squat down, then jump up. On landing, lower the body back into the squat. | 1 x 5 | 1 x10 | 1 x15 |
| Tuck-jump | Jump up, approaching the chest with bent knees. | 1 x 5 | 1 x10 | 1 x15 |
| High-step hop | Hop up on and down off a 5-inch-high step and switch between both feet. | 1 (5RT and 5LT) x 5 | 1 (5RT and 5LT) x 10 | 1 (5RT and 5LT) x 15 |
| High-step jump | Jump up on and down off a 5-inch-high step and switch between both feet. | 1 (5RT and 5LT) x 5 | 1 (5RT and 5LT) x 10 | 1 (5RT and 5LT) x 15 |
| - Blocks are presented as the number of sets/repetitions. - Children were asked to perform all repetitions successively with no pauses and encouraged to maximize their efforts (e.g., horizontal distance; vertical height; reduced ground contact time ) as much as possible. - Rest intervals of 1-2 minutes were allowed between exercise sets. - A preliminary test of children's performance was conducted in a sample of 5 children to determine the number of repetitions they should start within the first block. - Abbreviations: RT: right side, LT: left side. | | | | |
